# Household socio-economic position and individual infectious disease risk in rural Kenya

**DOI:** 10.1038/s41598-019-39375-z

**Published:** 2019-02-27

**Authors:** W. A. de Glanville, L. F. Thomas, E. A. J. Cook, B. M. de C. Bronsvoort, N. C. Wamae, S. Kariuki, E. M. Fèvre

**Affiliations:** 1 Centre for Immunity, Infection and Evolution, Institute for Immunology and Infection Research, School of Biological Sciences, University of Edinburgh, Ashworth Laboratories, West Mains Road, Edinburgh, EH9 3JT UK; 2 International Livestock Research Institute, Old Naivasha Road, PO BOX 30709, 00100 Nairobi, Kenya; 3 The Royal (Dick) School of Veterinary Studies, University of Edinburgh, Roslin, Midlothian, EH25 9RG UK; 4 Roslin Institute, The Royal (Dick) School of Veterinary Studies, University of Edinburgh, Roslin, Midlothian, EH25 9RG UK; 5 School of Pharmacy and Health Sciences, United States International University-Africa, PO Box 14634-01000, Nairobi, Kenya; 6 Centre for Microbiology Research, Kenya Medical Research Institute, PO Box 19464-00200, Nairobi, Kenya; 7 Institute of Infection and Global Health, University of Liverpool, Leahurst Campus, Neston, CH64 7TE UK; 8 Present Address: Institute of Biodiversity, Animal Health and Comparative Medicine, University of Glasgow, Glasgow, G12 8QQ United Kingdom

## Abstract

The importance of household socio-economic position (SEP) in shaping individual infectious disease risk is increasingly recognised, particularly in low income settings. However, few studies have measured the extent to which this association is consistent for the range of pathogens that are typically endemic among the rural poor in the tropics. This cross-sectional study assessed the relationship between SEP and human infection within a single community in western Kenya using a set of pathogens with diverse transmission routes. The relationships between household SEP and individual infection with *Plasmodium falciparum*, hookworm (*Ancylostoma duodenale* and/or *Necator americanus*), *Entamoeba histolytica/dispar*, *Mycobacterium tuberculosis*, and HIV, and co-infections between hookworm, *P. falciparum* and *E. histolytica*/*dispar*, were assessed using multivariable logistic and multinomial regression. Individuals in households with the lowest SEP were at greatest risk of infection with *P. falciparum*, hookworm and *E. histolytica/dispar*, as well as co-infection with each pathogen. Infection with *M. tuberculosis*, by contrast, was most likely in individuals living in households with the highest SEP. There was no evidence of a relationship between individual HIV infection and household SEP. We demonstrate the existence of a household socio-economic gradient within a rural farming community in Kenya which impacts upon individual infectious disease risk. Structural adjustments that seek to reduce poverty, and therefore the socio-economic inequalities that exist in this community, would be expected to substantially reduce overall infectious disease burden. However, policy makers and researchers should be aware that heterogeneous relationships can exist between household SEP and infection risk for different pathogens in low income settings.

## Introduction

More than one billion people live on less than 1.25 US dollars per day^1^. People in these circumstances typically live in communities where inadequate sanitation, limited access to health care and under-nutrition are widespread^2^. This structural poverty promotes the transmission and persistence of a wide range of infectious diseases^3^. The neglected tropical diseases are known to cluster and overlap within such communities, where they typically co-occur with HIV, TB and malaria^4^. Important health inequalities can exist within many of these impoverished communities: individuals living in households with the lowest socio-economic position (SEP) have been shown to be at greatest risk of infection for a range of pathogens^5–8^. The effect of a socioeconomic gradient on infectious disease risk, and poor health more broadly, has been observed even in communities where households may appear to the outside observer to be uniformly ‘poor’^9^. The relationship between a household’s SEP and the infection risk of its members is likely to be mediated through a wide range of factors, including the availability and use of sanitation, preventive and curative health care, and through unhygienic practices and behaviours^10^. Household poverty can therefore act as a common risk factor for a wide range of infectious agents, which may themselves interact to influence susceptibility to co-infecting pathogens and overall disease severity^11,12^, further burdening the poorest households.

To date, community-based observational studies quantifying the relationship between household SEP and infectious disease risk have tended to focus on single infectious outcomes, particularly HIV^13^, TB^14–16^, malaria^8,17,18^, and intestinal helminths^5^. A smaller number have included household SEP as a predictor of helminth-malaria co-infection^19,20^, or gastrointestinal parasitism with a number of similar species^6,7^. Few studies have explored the importance of household SEP as a shared risk factor for the wide range of infectious agents that are typically endemic in low income settings in the tropics^21^. The aim of this study was to quantify the extent to which household SEP could be considered a common risk factor for infection with multiple pathogens in a single community. The study was conducted in a rural area of Kenya known to be heavily burdened with a number of endemic infectious diseases^21^, and which is characterised by high levels of household poverty.

## Results

**Figure 1:**
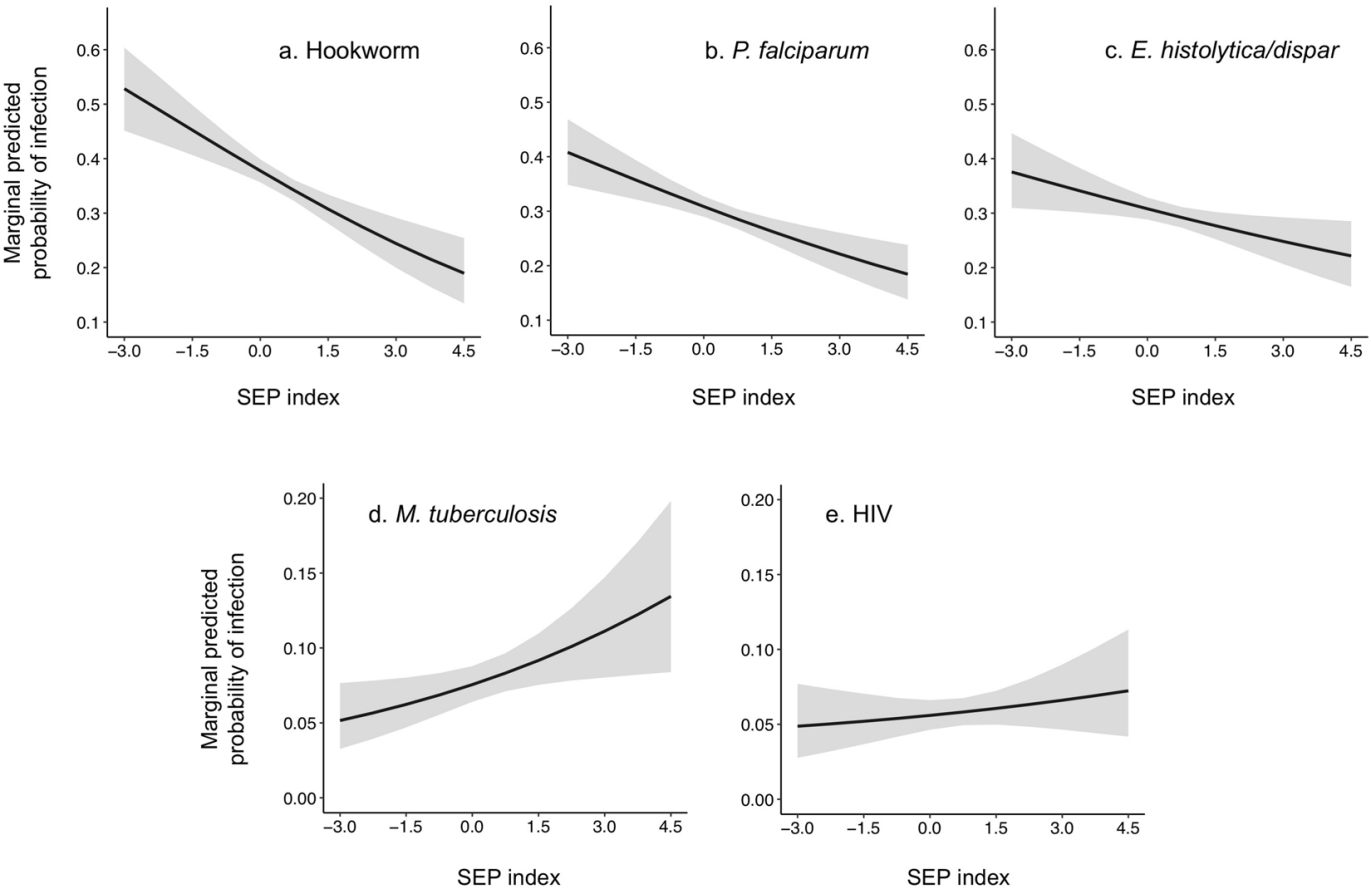
Posterior average marginal predicted probabilities of individual infection across the range of values of SEP. Grey areas represent 95% credibility intervals.

The unadjusted prevalence of individual-level infection with the soil-transmitted helminth, hookworm (due to *Ancylostoma duodenale* and/or *Necator americanus*) in the community under study was 35.5% (95% confidence interval (CI) 33.4–37.6); 29.9% (95% CI 27.9–31.9) for infection with the water-borne protozoan, *Entamoeba histolytica/dispar;* 29.7% (95% CI 27.8–31.7) for infection with the mosquito-borne malaria parasite, *Plasmodium falciparum*; 8.0% (95% CI 6.8–9.3) for infection with species in the *Mycobacterium tuberculosis* complex; and 5.9% (95% CI 4.9–7.0) for infection with HIV. On the basis of a multivariable logistic regression model with adjustment for a range of demographic and environmental variables operating at the individual and household level, there was evidence that the probability of individual infection with hookworm, *P. falciparum* and *E. histolytica/dispar* decreased as the SEP of the household to which individuals belong increased. An individual in the poorest household was predicted to have an average probability of infection with hookworm of 0.53 (95% credibility interval (CrI) 0.45–0.60) whilst this was 0.19 (95% CrI 0.13–0.25) in the richest household (Fig. 1). Similarly, a person in the poorest household was predicted to have an average probability of infection with *P. falciparum* of 0.41 (95% CrI 0.35–0.47) compared to 0.18 (95% CrI 0.14–0.24) in the richest. This was 0.38 (95% CrI 0.31–0.45) compared to 0.22 (95% CrI 0.16–0.29) for *E. histolytica/dispar*. The reverse trend was observed for HIV and *M. tuberculosis*, with the probability of individual infection increasing as SEP increased, although there was little evidence to support this relationship for HIV (Table 1). Were all individuals in the community to have the same risk of infection as those people in the richest households, and all else being equal, the overall prevalence of individual infection with hookworm, *P. falciparum* and *E. histolytica/dispar* could be predicted to decline by around 47%, 39% and 26%, respectively.

**Table 1:** Posterior estimates from the multivariable logistic regression models for individual infection. Estimates in bold indicate predictions where the 95% credibility intervals (95% CrI) do not include one, and therefore provide strong evidence for the observed relationship

	Hookworm	*P. falciparum*	*E. histolytica*	*M. tuberculosis*	HIV
	OR (95% CrI)	OR (95% CrI)	OR (95% CrI)	OR (95% CrI)	OR (95% CrI)
SEP	**0.65 (0.54–0.78)**	**0.76 (0.66–0.86)**	**0.84 (0.72–0.97)**	**1.31 (1.08–1.62)**	1.11 (0.87–1.44)
Mean NDVI	**1.37 (1.14**–**1.67)**	1.03 (0.90–1.18)	1.12 (0.97–1.30)		
Max.LST	1.16 (0.96–1.40)	1.12 (0.98–1.28)	1.12 (0.98 – 1.30		
Urban distance	1.01 (0.84–1.22)	**1.16 (1.01**–**1.33)**	0.93 (0.81–1.08)	0.89 (0.71– 1.11)	1.07 (0.83–1.38)
Luo household	**0.50 (0.32**–**0.78)**	1.29 (0.93–1.79)	0.86 (0.61–1.22)	1.34 (0.82–2.15)	**4.24 (2.43**–**7.72)**
Male	**1.45 (1.15**–**1.82)**	1.11 (0.89–1.38)	**0.75 (0.60**–**0.93)**	1.22 (0.85–1.77)	**0.53 (0.33**–**0.83)**
Age	**1.75 (1.51**–**2.12)**	**0.31 (0.26**–**0.36)**		**1.70 (1.42**–**2.04)**	**5.83 (3.88**–**9.11)**
Age × Age	**0.82 (0.73**–**0.92)**				**0.39 (0.29**–**0.50)**
5–4 years			*Ref*.		
15–24 years			**1.53 (1.14–2.05)**		
25 + years			1.01 (0.80–1.28)		
Hookworm EPG				1.10 (0.943–1.27)	
HIV infection				0.71 (0.29–1.57)	
**Random effects**					
${\sigma }_{{H}}^{2}$ (95% CrI)	1.79 (1.26–2.45)	0.50 (0.23–0.85)	0.79 (0.49–1.15)	0.64 (0.015–1.45)	1.40 (0.48–2.70)
PCV	9.96%	12.43%	3.54%	7.0%	−2.48%
POOR	41%	39%	44%	41%	48%

There was no evidence for confounding of the observed relationship between probability of infection and SEP for any pathogen. The univarable odds ratio (OR) for the effect of SEP on hookworm infection was 0.69 (95% CrI 0.58–0.82), 0.74 (95% CrI 0.66–0.84) for *P. falciparum*, 0.85 (95% CrI 0.74–0.98) for *E. histolytica/dispar* and 1.27 (95% CrI 1.06–1.52) for *M. tuberculosis*: none of these values were appreciably different from the multivariable estimates. Recent antimalarial use was protective against *P. falciparum* infection when included in the full multilevel logistic regression model (OR = 0.67, 95% CrI 0.46–0.96), however there was no mediation of the effect of household SEP on probability of infection: the OR for the effect of SEP on *P. falciparum* infection with control for antimalarial use was 0.76 (95% CrI 0.66–0.87).

Despite the strength of the individual-level associations observed, SEP explained only a small proportion of the between-household variation in individual risk for any infection (based on proportional change in variance (PCV), Table 1). The proportion of opposed odds ratios (POOR) were also moderately high for each pathogen, implying heterogeneous relationships with SEP exist between households. A POOR value of 0% would indicate the effect of SEP on individual infectious disease risk in all households is in the same direction, while a value of 50% would suggest individual disease risk in half of all households had the opposite relationship with SEP to the overall trend.

### Co-infection

Co-infection with multiple pathogens was common, with an unadjusted prevalence of 12.2% (95% CI 10.8–13.8) of individuals having concurrent infections with both hookworm and *E. histolytica/dispar*, 9.6% (95% CI 8.3–10.9) with both *P. falciparum* and *E. histolytica/dispar* and 10.7% (95% CI 9.4–12.2) with concurrent infections with hookworm and *P. falciparum*.

**Figure 2:**
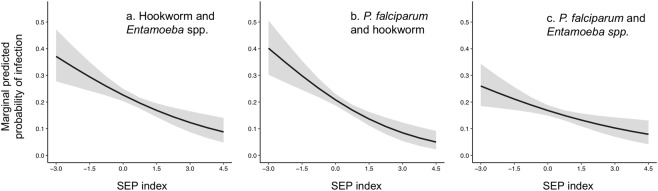
Posterior average marginal predicted probabilities of co-infection across the range of values of SEP. Grey areas represent 95% credibility intervals.

Table 2 gives the outputs from a logistic regression comparing co-infection with no infection and Fig. 2 gives the average marginal predicted probabilities for the effect of SEP on each pathogen pair. Increasing SEP reduced the probability of co-infection in each case, with an individual in the poorest household predicted to have an average probability of 0.37 (95% CrI 0.27–0.48) of being infected with both hookworm and *E. histolytica/dispar* whilst this was 0.092 (95% CrI 0.048–0.15) in the richest household. Similarly, an individual in the poorest household was predicted to have an average probability of infection with *P. falciparum* and *E. histolytica/dispar* of 0.26 (95% CrI 0.18–0.35) whilst this was 0.081 (95% CrI 0.042–0.14) in the richest household. For co-infection with *P. falciparum* and hookworm, this was 0.40 (95% CrI 0.29–0.51) in the poorest household compared to 0.055 (95% CrI 0.023–0.10) in the richest. All else being equal, if all individuals in the community had the same risk of co-infection with hookworm and *E. histolytica/dispar*, *P. falciparum* and *E. histolytica/dispar*, and *P. falciparum* and hookworm as those people in the richest households, the overall prevalence of co-infection in the whole community could be predicted to decline by around 25%, 16% and 49%, respectively.

**Table 2:** Posterior estimates from the multivariable logistic regression models comparing risk of co-infection with absence of infection with either parasite in a pair. Estimates in bold indicate predictions where the 95% credibility intervals (95% CrI) do not include one, and therefore provide strong evidence for the observed relationship.

	Hookworm/Entamoeba	Hookworm/malaria	Malaria/Entamoeba
	OR (95% CrI)	OR (95% CrI)	OR (95% CrI)
SEP	**0.60 (0.44**–**0.80)**	**0.45 (0.30**–**0.66)**	**0.69 (0.52**–**0.91)**
Mean NDVI	**1.53 (1.12**–**2.12)**	1.50 (1.00–2.31)	1.15 (0.87–1.52)
Maximum LST	1.33 (0.97–1.86)	1.65 (1.06–2.70)	**1.44 (1.03**–**2.09)**
Urban distance	0.97 (0.73–1.31)	1.18 (0.83–1.68)	1.15 (0.89–1.50)
Luo household	0.53 (0.26–1.06)	0.66 (0.27–1.60)	1.29 (0.69–2.47)
Male	1.06 (0.73–1.56)	**1.89 (1.19**–**3.04)**	0.89 (0.60–1.32)
Age	**1.34 (1.11**–**1.63)**	**0.41 (0.30**–**0.55)**	**0.26 (0.18**–**0.35)**
**Random effects**			
${\sigma }_{{H}}^{2}$ (95% CrI)	2.77 (1.65–4.27)	4.23 (2.45–6.67)	1.65 (0.74–2.90)
PCV	10.18%	16.98%	10.61%
POOR	34%	39%	42%

Socioeconomic position explained a moderately small amount of the between household variation in individual co-infection risk, and in all cases POOR values were large (Table 2). Household-level residual variation in individual co-infection risk was substantially larger than was the case for individual infection, suggesting important clustering of co-infection at the household level.

## Discussion

We demonstrate strong and significant relationships between SEP and individual infection risk in a rural population in Kenya. The community under study is characterised by high levels of poverty: Busia district, which covers the majority of the study area, was reported to be the 10^th^ poorest out of Kenya’s 70 districts^22^. The apparent homogeneity of households in the area, in which the majority of people live in dwellings constructed from local materials and have limited access to adequate sanitation, piped water or mains electricity^21^, could lead to the conclusion that such populations are relatively homogeneous in terms of their socioeconomic status^9^. However, we were able to identify a clear socioeconomic gradient in this community, and this gradient was significantly associated with risk of infection for a range of pathogens. The key finding from this study is therefore support for the general trend for a positive relationship between increasing SEP and health^23^, with evidence for reduced individual risk of infection with hookworm, *P. falciparum* and *E. histolytica/dispar* and co-infection with each pathogen as household SEP increases. However, this effect was not consistent for all pathogens, and we find evidence that the reverse effect exists for infection with *M. tuberculosis*, for which individuals in the wealthiest households appear to be at greatest risk.

Relationships between the environmentally- and vector-transmitted infectious agents and socioeconomic status are well known. Several studies have shown that the wealthiest individuals in endemic areas are least likely to have *P. falciparum* parasitaemia^8,17,24^, are more likely to use antimalarials^17,18^, and to seek them more rapidly^25^. The increased use of antimalarials by individuals in wealthier households may explain some of the observed effect on risk of infection^9^, although we found minimal mediation by antimalarial use. Other factors, such as sleeping under a bed net, outdoor activity or housing quality may be more important as mediators of infection. The relationship between SEP and hookworm infection was stronger than that for any other pathogen. Several factors may link household SEP to individual risk of infection, particularly the availability and use of latrines and therefore the level of larval contamination in the domestic and peri-domestic environment^5^. The effect of SEP on exposure to infectious hookworm larvae may be mediated through factors such as agricultural occupation, type of foot wear used, education, and household building materials^5^. The negative relationship we observed between increasing SEP and infection with *E. histolytica/dispar* has also been described previously^6,26^. These parasites are transmitted via the faecal-oral route, and the use of contaminated water sources, consumption of contaminated food or inadequate hygiene practices in the poorest households are likely to be important in mediating the effect observed^27^.

Boccia *et al*.^14^ report that infection with *M. tuberculosis* was most prevalent in the wealthiest individuals in a cross-sectional survey in Zambia. A similar effect was reported for active TB in a single population studied at different time points in Malawi^15,28^. These and our own findings suggest *M. tuberculosis* infection may not always follow the positive social gradient in health. Infection with HIV has been previously shown to be most prevalent in individuals with higher socioeconomic status in low income settings^13,29^. However, in our population HIV was not significantly related to SEP and did not affect the relationship between SEP and *M. tuberculosis* infection. Factors that link household SEP to increased *M. tuberculosis* infection may include individual smoking, alcohol consumption and behavioral factors that influence time spent in confined, poorly ventilated places such as bars, churches or public transport^14,15,28^. It is important to note that these findings relate only to infection with *M. tuberculosis*, and not clinical tuberculosis. Progression to clinical disease is known to be influenced by cofactors such as poor nutrition and co-infection^30,31^, which are strongly associated with poverty. The elimination of poverty and expansion of social protection is expected to substantially reduce the incidence in tuberculosis^32^, which is likely to influence the prevalence of individual infection with *M. tuberculosis*.

We provide evidence for the clustering of co-infection at the household-level, and a strong association between household SEP and co-infection, such that individuals in the poorest households are at greatest risk for multiple infections. This supports the notion of syndemics, or the population-level clustering of two or more diseases shaped by contextual and social factors^12^. It also points to the need for integrated interventions and structural changes to tackle the health inequalities that exist in this population. Such interventions could include social policies aimed at poverty alleviation and reducing barriers to health care, as well as disease-specific approaches, including increased access to sanitation and biomedical technologies such as water filtration, insecticide-treated bed nets and antimicrobials. The findings from this study suggest that were all households to have the material and productive assets, access to services and preventive health measures, and household resources of the wealthiest household in this community, the prevalence of individual hookworm, *P. falciparum* and *E. histolytica* and co-infection with each could be expected to decline substantially.

It should be noted that in no case did SEP explain a large amount of the between-household variation in individual risk, which was particularly large for parasite co-infection. This may be a limitation of the index defined, which can only be a representation of the relative social and economic position of a particular household^33^. It does suggest, however, that there are factors other than SEP that may also influence health inequalities in this community. These could include environmental conditions: hookworm in particular was strongly positively associated with normalised difference vegetation index (NDVI), a finding that has been reported previously^34^. Other social conditions may also be important. *Plasmodium falciparum* infection, for example, was positively related to increasing distance from an urban area, a finding that has also been reported elsewhere^35^. Luo ethnicity was negatively associated with hookworm infection in the multivariable model. We are not aware of an explanation for this effect, which warrants further explanation. This ethnic group also appears to be at substantially elevated risk for HIV infection, supporting previous findings in the study area^36^. The proportion of variation in infectious disease risk that SEP explains can also be expected to vary depending on characteristics of transmission within the community^37^. When considering transmission of the highly prevalent parasitic species included here, exposure to contaminated soil, water or food is likely to be a frequent occurrence outside an individual’s own household environment^10,38–40^. Moreover, while the majority of *P. falciparum* transmission is thought to occur in the domestic environment, outdoor biting mosquito vector species such as *Anopholes arabiensis* are common, and outdoor transmission known to be important in western Kenya^41^. Hence, individuals from relatively rich as well as relatively poor households may have high levels of exposure to pathogens such as hookworm, *E. histolytica/dispar* and *P. falciparum* within the “public domain”^10^. Changes in community-level prevalence, or structural improvements that reduce transmission in the community, could therefore be expected to influence the relative contribution of the “domestic domain”, and household SEP in particular, in structuring individual infection risk for these parasitic diseases.

## Methods

Data were collected as part of the ‘People, Animals and their Zoonoses’ (PAZ) study. The study and its methods have been described in detail previously^21^. Briefly, this was a large cross-sectional survey of 416 randomly selected households in a mixed farming community in Bungoma, Siaya, Kakamega and Busia counties in western Kenya. Households were randomly selected from within sub-locations, with the number of households (between 1 and 8) selected per sublocation proportional to the cattle population (since the primary focus for the PAZ study was on zoonotic disease). In total, 2113 consenting individuals ≥5 years of age were sampled between September 2010 and July 2012 and tested for recent exposure or current infection with a wide range of pathogens.

### Classifying outcomes

We have previously reported the prevalence for a number of human pathogens in this community^21^, and here we focus on those that are prevalent, represent a range of transmission routes, and are known to have important burdens within low income communities. These were the soil transmitted helminths, *Ancylostoma duodenale* and/or *Necator americanus*, hereafter referred to as hookworm; *Entamoeba histolytica/dispar; Plasmodium falciparum*; species in the *Mycobacterium tuberculosis* complex; and HIV. Given the hypothesised shared effect of household poverty, we also explored relationships between SEP and individual-level co-infection with pairs of the pathogens described.

Individuals were classified as infected with *P. falciparum* when parasites were identified on thick or thin blood smears stained with Giemsa using light microscopy. Hookworm infection was defined as the presence of at least one egg in a single faecal sample examined using either using the Kato-Katz (KK) and formal ether concentration (FEC) techniques^42,43^. Quantification of the number of eggs per gram (EPG) of faeces was conducted using standard techniques^42,43^. Infection with *E. histolytica/dispar* was assigned based on identification of at least one cyst in a single faecal sample prepared using the FEC technique. Infection with *M. tuberculosis* was determined using a gamma-interferon assay (QuantiFERON-TB test, Cellestis Limited). HIV infection was defined using a rapid strip test (SD Bioline HIV 1/2 3.0, Standard Diagnostics)

Sampled individuals were nested within households. These represent patrilineal family groups living within a single compound of multiple dwellings. The average reported household size was 7.6 (range 1 to 30) people, from which our average household sample size was 5.1 (range 1 to 21)^21^.

### Ethical approval

Ethical approval for this study was granted by the Kenya Medical Research Institute (KEMRI) Ethical Review Board (SCC1701). All activities were conducted in accordance with protocols approved by this review board. All participants and/or their legal guardians provided written informed consent.

### Index of household socioeconomic position

**Figure 3:**
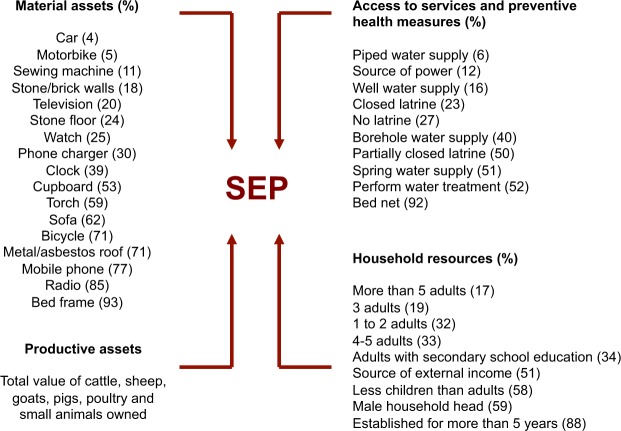
Groups of variables considered to influence household SEP. Numbers in parentheses represent the percentage of households in the study area reporting each binary variable.

A questionnaire conducted with the head of the household was used to collect data on a set of variables expected to provide information on socio-economic position (SEP), or the “social and economic factors that influence the position individuals or groups hold within the structure of society”^44^. These variables fell into four groups: 1) material assets; 2) productive assets; 3) access to services and preventive health measures; and 4) household resources (Fig. 3) (s*ensu* Boccia *et al*.^14^).

Given these natural groupings of variables, we used multiple factor analysis (MFA) to derive an index of household SEP. The MFA was performed in R statistical environment (version 3.1.1) using the package *FactoMineR*^45^. The productive asset domain was represented by household total livestock value (TLV) and was derived using average estimates of the monetary value for each class and age of animal owned by livestock keeping households. Livestock values were gathered from key informants at local markets and slaughterhouses. Household TLV ranged from 0 to £1593, with a median of £75 and mean of £155^46^. Multiple factor analysis assumes that there is an underlying causal structure in the groupings of variables chosen, and that the co-variation observed is due to the presence of one or more latent variables (the factors) that exert a causal influence on the observed variables^47^. We used the first factor derived from the MFA, which captures the most variation in the component variables, to represent this underlying household SEP variable.

The full set of scores assigned to each variable used to derive the index, and their relative contribution, is given in the supplementary materials.

### Relationship between SEP and infection risk

Associations between infection and SEP were examined using multilevel logistic regression. Since the main relationship of interest was between household SEP and probability of infection, we fit full models containing SEP and the set of covariates considered *a priori* to be potential confounders. For the parasitic infections (*P. falciparum*, hookworm and *E. histolytica/dispar*) covariates were individual age and sex, and household-level measures of mean normalised difference vegetation index (NDVI), maximum land surface temperature (LST), distance to an urban area, and household membership of the Luo ethnic group. For *M. tuberculosis* and HIV, only distance to an urban area and household Luo ethnicity were included with age and sex.

‘Urban’ was defined as an area with a population density greater than the 99^th^ percentile for the study area, with data from http://www.worldpop.org.uk/. Fourier processed MODIS mean NDVI and maximum LST at 1 km resolution were obtained for the study area from Scharlemann *et al*.^48^. Data were manipulated and extracted at the household level in ArcMap 10.1 (ESRI 2012, Redlands). Household Luo ethnicity was assigned when 50% or more of the adults reported being Luo. We focused on membership of this ethnic group because it is known to be highly geographically localised in the southern part of the study area^21^, and has previously been shown to be heavily burdened by HIV infection in western Kenya^36^.

Full multilevel logistic regression models were fit in WinBUGS (MRC Biostatistics Unit, Cambridge, UK) using weakly informative normal priors for all fixed and random effects. The precision for household level random effects was defined using a wide uniform hyperprior (i.e. Uniform(0,100)). Model convergence was confirmed by visual assessment of MCMC chains. Inference was based on 3 chains that were allowed to run for at least 70,000 iterations after a burn-in of at least 30,000 and a thinning interval of 10. For each outcome, we present odds ratios and average marginal predicted probabilities of infection which were estimated across all households and all individuals at a range of values of SEP. All continuous covariates were centred and standardised with a mean of zero and standard deviation of one to assist model convergence. Relationships between each pathogen and continuous covariates were assumed to be linear, except for age where improvements in model fit were examined using quadratic or categorical specifications.

### Mediation by access to health care

Potential mediation of the effect of SEP on *P. falciparum* infection by recent antimalarial use was examined using a hierarchical approach^49^. For this, the reported recent use of anti-malarials (in the past 4 weeks) was included in the full model for *P. falciparum* and the change in the co-efficient for the effect of SEP determined.

### Co-infections

The Begg and Gray approximation^50^ for multinomial logistic regression was used to quantify the relationship between SEP and individual level co-infections. Pathogen pairs considered were *P. falciparum* and hookworm; *E. histolytica/dispar* and hookworm; and *P. falciparum* and *E. histolytica/dispar*. In each case, the nominal outcome was defined as: 1) no infection with either pathogen and 2) infection with both pathogens. Multivariable models were fit with control for age, sex, mean NDVI, maximum LST, distance to an urban area and household Luo ethnicity as fixed effects and household as a random effect in WinBUGS using the settings described above.

Infection with HIV and *M. tuberculosis* was comparatively rarer in the study area than infection with the parasitic infections, and we did not explore co-infections with these and each of the other pathogens. However, helminth infection has been shown to suppress the Th1 mediated mechanisms that may determine IFN-gamma production in response to exposure to species in the *M. tuberculosis* complex^51^. This is the immunological process measured by the gamma interferon assay, and helminth infection could therefore be expected to confound the observed relationship between *M. tuberculosis* and SEP. Hookworm eggs per gram (EPG) of faeces was included as a fixed effect in the full model for *M. tuberculosis* infection to control for this potential confounding. Positive associations between HIV and TB have also been widely described^52^, and the former could confound the relationship of the latter with SEP. Infection with HIV was therefore also included as a fixed effect in the multivariable logistic regression for *M. tuberculosis* infection.

### Household contextual effects

In order to explore the proportion of between-household variation in individual risk that SEP explained, we compared the full multilevel model for each outcome with a reduced model without SEP. The proportional change in variance (PCV) was calculated as^53^:

$${\rm{PCV}}=\,\frac{{V}_{A}-{V}_{B}}{{V}_{A}}\times 100$$

where V_A_ is the household-level variance of the reduced model without SEP and V_B_ the household-level variance of the full model.

To further quantify the household-level effect of SEP in explaining individual variation in infection risk, we derived the proportion of opposed odds ratios (POOR) statistic. This allowed the integration of the fixed effect of SEP with the random residual variation in the log odds of infection for each pathogen or pathogen pair between households. The POOR is calculated as^54^:

$$POOR={\rm{\Phi }}[-|\frac{\beta }{\sqrt{2{\hat{\tau }}^{2}}}|]\,$$

where *β* is the regression co-efficient for the group effect (SEP) and ${\hat{\tau }}^{2}$ the variance of the distribution of group- (household) level random effects. The POOR can take any value from 0 to 50% and estimates the proportion of group-level odds ratios (OR) that have the opposite sign to the overall OR (indicated by *β*)^54^.

## Supplementary information

Supplementary materials

## Data Availability

Anonymised data are available at 10.17638/datacat.liverpool.ac.uk/447.
