# Supplementary material for: Household socio-economic position and individual infectious disease risk in rural Kenya
Source: Sci Rep. 2019 Feb 27;9:2972. doi: 10.1038/s41598-019-39375-z (PMC6393457; doi:10.1038/s41598-019-39375-z)
Supplement: Supplementary file 1 — Supplementary materials [file 41598_2019_39375_MOESM1_ESM.doc]

**Supplementary materials**

**“Household socio-economic position and individual infectious disease risk in rural Kenya”**

**de Glanville W.A., Thomas L.F., Cook E.A.J., Bronsvoort B.M., Wamae N.C., Kariuki S., Fèvre E.M.**

**Scores and contributions of variables to the MFA**

The scores (i.e. principal co-ordinates) and the relative contribution (i.e. the quotient between the inertia of a point’s projection and the inertia of all points on a particular axis, in this case the first) assigned to each variable in the MFA is shown in Supplementary Table 1. The average household SEP value was 0, and therefore variables which have a negative score can be considered to make a negative contribution to SEP, whilst those with a positive score have a positive effect (i.e. a household has a higher SEP if it possesses them). In the case of material wealth, every asset owned has a positive effect on wealth, whilst not owning the asset makes a negative contribution. Similarly, not having access to household services tends to result in a negative score, whilst access indicates higher SEP. The effect of variables in the household resources group is also as expected, with the higher number of adults, adult education and an external source of income all making positive contributions to household SEP.

The output from the MFA is further summarised in Supplementary Figure 1. The position of the greyed points along the x axis represent the SEP score assigned to each household under study. There was no evidence of ‘clumping’, which may occur when household scores are distributed into identifiable clusters (e.g. due to differences between urban and rural), or of ‘truncation’, where scores are spread over a narrow range.^1^ The weights (scores, or principal co-ordinates) assigned to those variables that made a large contribution to the derivation of the index (i.e. contribution to inertia of the axis) are also presented.

**Supplementary Table 1.** Variable scores and relative contributions to the final MFA

| **Material wealth** | **Scores** | **Contrib.** | **Access to services** | **Scores** | **Contrib.** |
| --- | --- | --- | --- | --- | --- |
| Cement floor | 1.43 | 2.77 | Power source | 2.2 | 7.88 |
| TV | 1.58 | 2.67 | Closed latrine | 1.28 | 5.4 |
| Brick/cement walls | 1.52 | 2.31 | No latrine | -1.06 | 5.06 |
| No sofa | -1.01 | 2.25 | No water treatment | -0.53 | 1.73 |
| No cupboard | -0.89 | 2.08 | No bed net | -1.15 | 1.57 |
| No mobile phone | -1.26 | 2.04 | Water treatment | 0.37 | 1.21 |
| Mobile phone charger | 1.12 | 1.98 | No power source | -0.27 | 0.96 |
| Cupboard | 0.79 | 1.86 | Use piped water | 0.81 | 0.87 |
| Thatched roof | -1.01 | 1.78 | Use borehole water | 0.25 | 0.41 |
| Sofa | 0.65 | 1.45 | Does not use borehole water | -0.2 | 0.34 |
| Clock | 0.82 | 1.44 | Use well water | 0.3 | 0.23 |
| No bicycle | -0.94 | 1.42 | Use spring water | -0.16 | 0.18 |
| No radio | -1.26 | 1.42 | Bed net | 0.1 | 0.14 |
| No torch | -0.74 | 1.22 | Does not use spring water | 0.13 | 0.14 |
| Sewing machine | 1.38 | 1.21 | Does not use piped water | -0.08 | 0.08 |
| Watch | 0.91 | 1.03 | Does not use well water | -0.06 | 0.04 |
| Mud floor | -0.46 | 0.89 | Partially closed latrine | 0.08 | 0.04 |
| No clock | -0.51 | 0.89 | **Household resources** | **Scores** | **Contrib.** |
| Torch | 0.49 | 0.81 | Adults with sec. school ed. | 1.04 | 4.92 |
| Metal roof | 0.46 | 0.8 | 1-2 adults in household | -0.95 | 4.33 |
| No mobile phone charger | -0.44 | 0.79 | >5 adults in household | 1.06 | 2.54 |
| Motorbike | 1.48 | 0.65 | No adults with sec. school ed. | -0.51 | 2.42 |
| No TV | -0.37 | 0.63 | External source of income | 0.58 | 2.27 |
| Mobile phone | 0.38 | 0.61 | No external source of income | -0.55 | 2.13 |
| No bed | -1.13 | 0.56 | 4-5 adults in household | 0.46 | 0.87 |
| Bicycle | 0.38 | 0.56 | Adults<Children | -0.41 | 0.85 |
| Mud walls | -0.33 | 0.5 | Adults> Children | 0.25 | 0.52 |
| No watch | -0.26 | 0.29 | Female household head | -0.19 | 0.2 |
| Radio | 0.24 | 0.27 | Male household head | 0.14 | 0.15 |
| No sewing machine | -0.18 | 0.15 | Household established <5yrs | -0.2 | 0.06 |
| Bed | 0.1 | 0.05 | 3 adults in household | 0.1 | 0.03 |
| No motorbike | -0.08 | 0.04 | Household established >5yrs | 0.03 | 0.01 |
| **Total livestock value** | **Scores** | **Contrib.** |  |  |  |
| TLV | 0.55 | 15.02 |  |  |  |

**
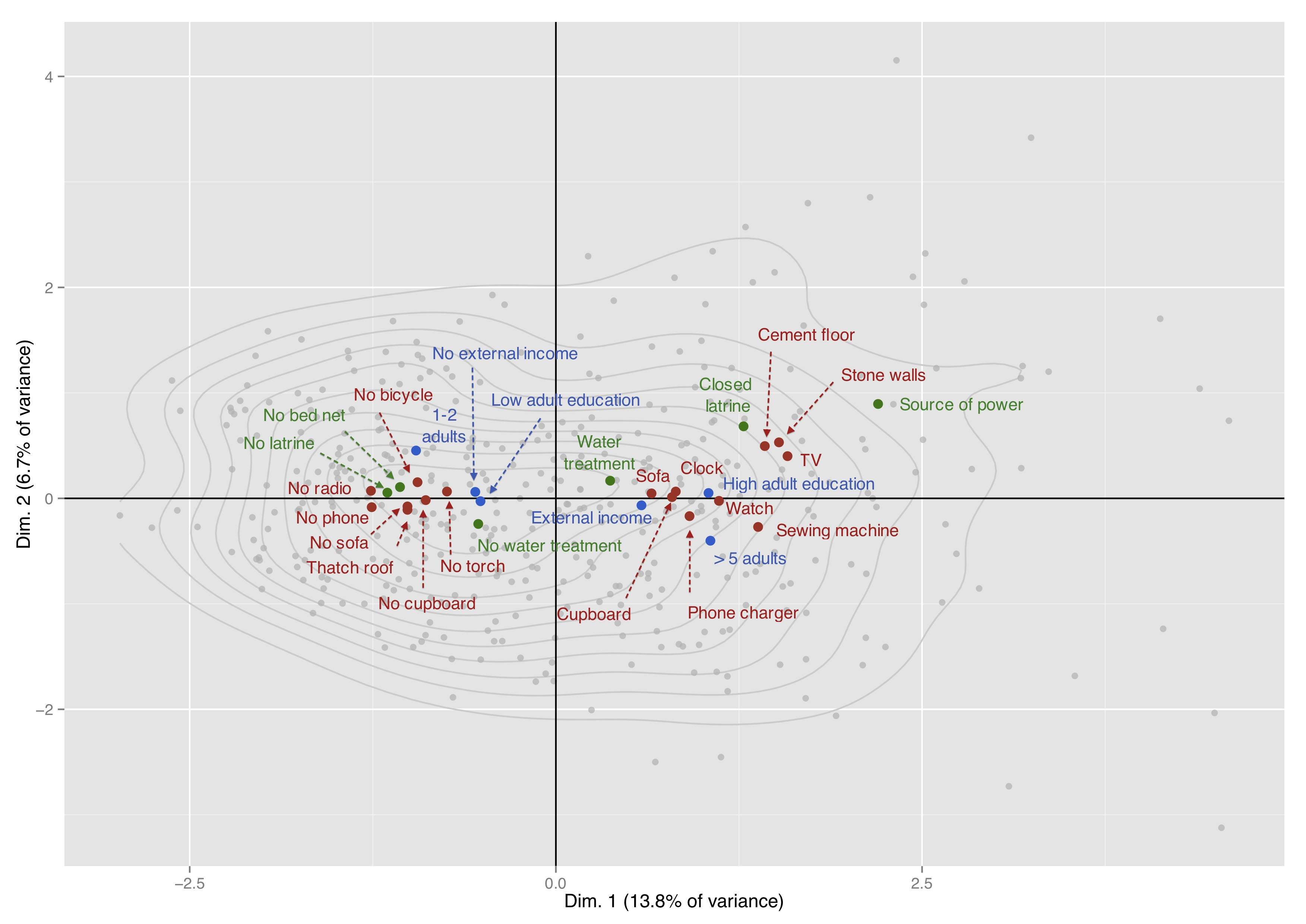
**

**Supplementary Figure 1**. **Scores of those variables which make a contribution to the inertia of first principal component (Dim. 1) greater than 1.** Red variable names are in the material wealth group; green in the access to services group; blue in the household resources group. Grey dots represent the score assigned to households.

**References**

1 Vyas S, Kumaranayake L. Constructing socio-economic status indices: how to use principal components analysis*. Health Policy Pl*an**;** 21: 459–68 (2006).
